# Electromagnetic sensing and infiltration measurements to evaluate turfgrass salinity and reclamation

**DOI:** 10.1038/s41598-022-09189-7

**Published:** 2022-03-24

**Authors:** Gülüzar Duygu Semiz, Donald L. Suarez, Scott M. Lesch

**Affiliations:** 1 Present Address: Department of Agricultural Structures and Irrigation, Agricultural Faculty, Ankara University, Ankara, Turkey; 2 USDA-ARS Salinity Laboratory, Riverside, CA USA; 3 City of Riverside, Public Utilities-Resource Operations & Strategic Analytics, Riverside, CA USA

**Keywords:** Geochemistry, Geochemistry, Environmental impact, Grassland ecology

## Abstract

Scarce freshwater resources in arid and semiarid regions means that recreational landscapes should use recycled or low-quality waters for irrigation, increasing the risk of salinity and infiltration problems. We map salinity distribution within turf fields using electromagnetic sensing, evaluate need for leaching and evaluate post leaching results for subsequent management decisions. Electromagnetic measurements were made with two EM38 instruments positioned vertically and horizontally in order to determine salinity distribution. Sensor readings were coupled to GPS data to create spatial salinity maps. Next, optimal calibration point coordinates were determined via Electrical Conductivity Sampling Assessment and Prediction (ESAP) software. Soil samples from 0–15 and 15–30 cm depths were used for each calibration point. Laboratory soil saturation percentage, moisture content, electrical conductivity (EC_e_) and pH_e_ of saturation extracts were determined for calibration to convert resistivity measurements to EC_e_. Next, EC_e_ maps were created using ESAP software. Leaching for reclamation was performed by means of sprinkling. Treated municipal wastewater was utilized both for irrigation and for reclamation leaching. Low water content and high spatial variability of soil texture adversely affected the accuracy of the readings. Pre and post leaching surveys indicate that in one fairway there was only a 43% and 58% decrease in soil salinity at 0–15 and 15–30 cm depths, respectively which is very low relative to expected results considering the amount of water applied. This relatively low reduction in salinity and the lack of runoff during irrigation combined with infiltration measurements suggests that aeration techniques for healthier grasses led to water bypassing small pores thus limiting leaching efficiency. In this instance practices to improve infiltration lead paradoxically to less salinity reclamation than expected.

## Introduction

Scarce freshwater supplies in arid and semi-arid regions means that whenever possible more saline lower quality waters should be used in agriculture, saving fresh water for municipal use. This substitution of lower quality water is especially feasible when biomass yield is not important, such as with recreational turfgrass. Use of these waters requires careful monitoring of salinity. Assessment of soil salinity has been determined in a number of ways: (i) plant observations, (ii) the electrical conductance of soil solution extracted via soil water extractors (iii) the electrical conductivity (EC) of water obtained by collecting soil samples, adding varying amounts of water to each sample and then extracting, (iv) in situ measurement of electrical resistivity (ER), (v) non-invasive determination of EC with electromagnetic induction (EMI), and most recently (vi) in situ determination of EC with time domain reflectometry (TDR). The techniques of ER, EMI, and TDR measure bulk soil (combined solid and liquid phase) electrical conductance, termed EC_a_^1^.

Compared to other methods, EMI measurements generate both spatially and temporally highly efficient data^2^. However, in contrast to the other methods EMI and ER have the disadvantage of requiring calibration to convert the soil EC_a_ data to soil water or saturation extract EC (EC_e_).

In the last 20 years, scientists have made advances to better understand the best application procedure for measuring soil salinity, water in soil pores, soil texture and depth etc^3–8^. By identifying the complexities of EC_a_ measurement and how to deal with them, Corwin and Lesch^9^ provided guidelines for the use of EC_a_ in agriculture. EM38 is a tool that measures EC_a_ value to a depth of approximately 0.75–1.5 m depth of the soil profile, depending on its horizontal and vertical positioning. The signal response is weighted to shallow depths, with an exponential decay in signal response from the surface to 0.75–1.5 m depth. This device consists of a receiver and a transmitting coil placed at opposite ends of the non-conductive rod at 1 m intervals^10^. The EMI technique produces readings on any conductive material as EC_a_, EC_a_ readings can be influenced not only by soil water salinity, but also by any property that affects soil conductivity such as soil water content and clay content. Calibration equations between EC_a_ and EC_e_ should be established considering site-specific properties such as soil texture, soil water content, soil salinity etc. in an area measured by the EM technique. Three parallel pathways of current flow contribute to the EC_a_ measurement: (1) a liquid phase pathway via dissolved salts contained in the soil water present in medium to large pores, (2) a solid phase pathway via conduction through soil particles that are in continuous, direct contact with one another, and (3) a solid–liquid pathway via hydrated exchangeable cations associated with clay minerals^11^.

Remote sensing has made the concept of precision agriculture practical. Precision agriculture utilizes detailed maps of soil or vegetative characteristics for site specific management^12^. Among the soil properties and characteristics of most interest are water content, soil texture and salinity. Precision agriculture has been applied to landscape vegetation as well as agriculture. Carrow et al.^13^ used the term precision turfgrass management (PTA) for application of the site-specific mapping concepts to turfgrass management of irrigation, salinity and fertilizer application and cultivation, utilizing both soil sampling and various field-based sensors. Devitt et al.^14^, evaluated the spatial and temporal changes in salinity on turfgrass using in situ sensors. This approach provides more accurate data than that obtained via remote sensing but has limited practicality due to cost of installation and need for many sensors to characterize variability.

To evaluate soil salinity, we are interested primarily in the electrical conductance of the soil solution. The EC_a_ measurement includes more than just soil salinity, as it is a measure of the sum of all conductive materials within the volume of measurement and is thus influenced by any soil property that affects bulk soil electrical conductance^13^. Use of a EC_a_ survey to measure salinity within an area has been divided into eight steps: (1) design for the EC_a_ survey, (2) spatially identified EC_a_ data collection (3) soil sampling design based on the spatial variations in the EC_a_ data (4) collection of soil samples at the identified optimal sites for EC_a_ calibration (5) physical and chemical analysis of relevant soil properties, primarily EC of a soil water extract, (6) spatial statistical analysis (7) determination of main soil properties in the study area affecting the EC_a_ measurements (8) GIS application^15^. The U.S. Salinity Laboratory Staff (ARS-USDA, Riverside, California) developed conductivity modeling software (Electrical conductivity Sampling Assessment and Prediction-ESAP)^16–20^ to promote efficient acquisition and construction of EC_a_ data. ESAP, user-friendly software, provides (i) survey maps and directed sample design relying on the maps (ii) calibration of EC_a_ readings to EC_e_ (iii) explication of estimated spatial salinity data. Acquired information is practical for salinity management^20^.

The aim of this study is to map the salinity distribution of two fairways at Dove Canyon golf course in Trabuco Canyon, California by means of EM38 sensors in order to determine need for reclamation and then remap the fairways to evaluate the success of attempted soil remediation via leaching. This report represents a use of the EM technique to enable faster diagnosis of salinity in golf courses with minimal detrimental soil disturbance from soil coring, critical to recreational turf.

## Results

### Leaching

The reference crop evapotranspiration (ET_0_) during July was 5.55 mm day^−1^ (CMIS station 75) with regular irrigation application averaging 3.9 mm day^−1^. Water application was thus 70% of potential ET. Under irrigation would result in slower growth and reduced plant density but by itself does not explain the poor turf status observed at the start of the study.

#### Soil samples, fairway 16

The salinity values for the soil cores taken in fairway 16 (Table 1) show a wide range in EC, varying from EC_e_ 5.68 to 20.5 dS m^−1^ in the 0–15 cm depth and from EC_e_ 2.5 to 36.1 dS m^−1^ in the 15–30 cm samples. Mean salinity level for the soil samples collected from fairway 16 before leaching was EC_e_ 12.27 and 11.83 dS m^−1^ for 0–15 and 15–30 cm depth respectively. These samples can be considered biased in that the sample locations were not random but rather selected by the ESAP software to capture the variability observed in the EM_a_ maps.

**Table 1:** Soil salinity at the sampling points for pre and post leaching for fairway 16.

Sample point	Depth (cm)	Pre leaching EC_e_ (dS m^−1^)	Post leaching EC_e_ (dS m^−1^)	Sample point	Depth (cm)	Pre leaching EC_e_ (dS m^−1^)	Post leaching EC_e_ (dS m^−1^)
18	0–15	5.68	7.28	164	0–15	18.8	9.35
	15–30	10.90	2.39		15–30	9.9	2.66
57	0–15	20.5	5.11	263	0–15	10.43	6.44
	15–30	10.3	11.57		15–30	8.55	7.19
87	0–15	6.7	1.93	268	0–15	6.27	3.34
	15–30	2.5	1.36		15–30	6.96	2.05
155	0–15	13.2	8.84	350	0–15	16.58	13.53
	15–30	9.4	1.26		15–30	36.10	11.32

Sites 110 and 217 could not be analyzed (no-pre leaching samples), due to insufficient residual soil volumes (for analysis).

After leaching the mean salinity level for fairway 16 was 6.98 and 4.98 dS m^−1^ for 0–15 and 15–30 cm respectively (based on data in Table 1). The reduction in average soil salinity is statistically significant p < 0.05. The salinity based on soil cores decreased by 43% for the 0–15 cm depth and by 58% for the 15–30 cm depth after application of 153 mm of water.

#### Soil samples, fairway 12

The EC_e_ results of the soil samples for fairway 12, pre leaching are given in Table 2. The salinity values show a wide range in EC, varying from EC_e_ 2.37 to 19.52 dS m^−1^ in the 0–15 cm depth and from EC_e_ 1.22 to 15.13 dS m^−1^ in the 15–30 cm samples. Mean salinity for the soil samples collected from fairway 12 before leaching were EC_e_ 8.46 and 6.32 dS m^−1^ for 0–15 and 15–30 cm depth respectively. Material below this depth consisted of relatively unweathered rock and was not collected.

**Table 2:** Soil salinity at the sampling points for pre and post leaching for fairway 12.

Sample point	Depth (cm)	Pre leaching EC_e_ (dS m^−1^)	Post leaching EC_e_ (dS m^−1^)	Sample point	Depth (cm)	Pre-leaching EC_e_ (dS m^−1^)	Post leaching EC_e_ (dS m^−1^)
11	0–15	10.47	13.90	214	0–15	13.27	8.88
	15–30	8.66	9.22		15–30	6.28	2.93
47	0–15	19.52	4.81	218	0–15	2.37	3.93
	15–30	4.90	4.78		15–30	2.35	2.43
94	0–15	6.62	2.50	279	0–15	13.82	2.13
	15–30	15.13	5.65		15–30	10.98	1.32
168	0–15	9.54	2.66	303	0–15	2.7	5.59
	15–30	9.07	2.27		15–30	2.4	2.26
188	0–15	2.94	5.33	345	0–15	3.32	3.21
	15–30	1.22	5.9		15–30	2.22	3.18

The mean salinity levels (EC_e_) based on soil cores for fairway 12 after leaching were 5.29 dS m^−1^ for the 0–15 cm depth and 3.99 for the 15–30 cm depth. The 37.5% and 36.9% reduction in 0–15 cm and 15–30 cm average soil salinity was statistically significant (p < 0.05).

### Field survey and salinity mapping

The EC_e_ results of the soil samples for both pre- and post-leaching of fairway 16, given in Table 1 and the results from fairway 12 in Table 2 were used to calibrate the EM-38 data (EC_a_ readings) for conversion into EC_e_ maps. We used a standard multiple linear regression (MLR) model defined as below.

1 $${\text{ln }}\left( {{\text{EC}}_{{\text{e}}} } \right): \, \beta_{0} + \beta_{{1}} {\text{ln }}\left( {{\text{EM}}_{{\text{v}}} } \right) + \, \beta_{{2}} {\text{ln }}\left( {{\text{EM}}_{{\text{h}}} } \right),$$

where EC_e_ is the soil salinity, EM_v_ and EM_h_ are the vertical and horizontal reading respectively, and β_0_, β_1_, and β_2_ are model parameters. Data were further corrected for variations in water content based on water content of the soil samples via ESAP.

Fairway 16 pre and post leaching EM38 data exhibit very high spatial consistency. Post leaching average EM levels increase slightly relative to pre leaching values (EM_v_: 77.5 to 80.0 mS m^−1^; EM_h_: 62.6 to 72.3 mS m^−1^). Both apparent reductions in EM readings are statistically significant (p < 0.05). The 15–30 cm salinity appears lower than the 0–15 cm salinity levels.

The median pre-leaching EC_e_ values for fairway 16 based on the EM reading converted to EC_e_ were 12.5 dS m^−1^ for 0–15 cm depth and 8.5 dS m^−1^ for 15–30 cm depth.

**Figure 1:**
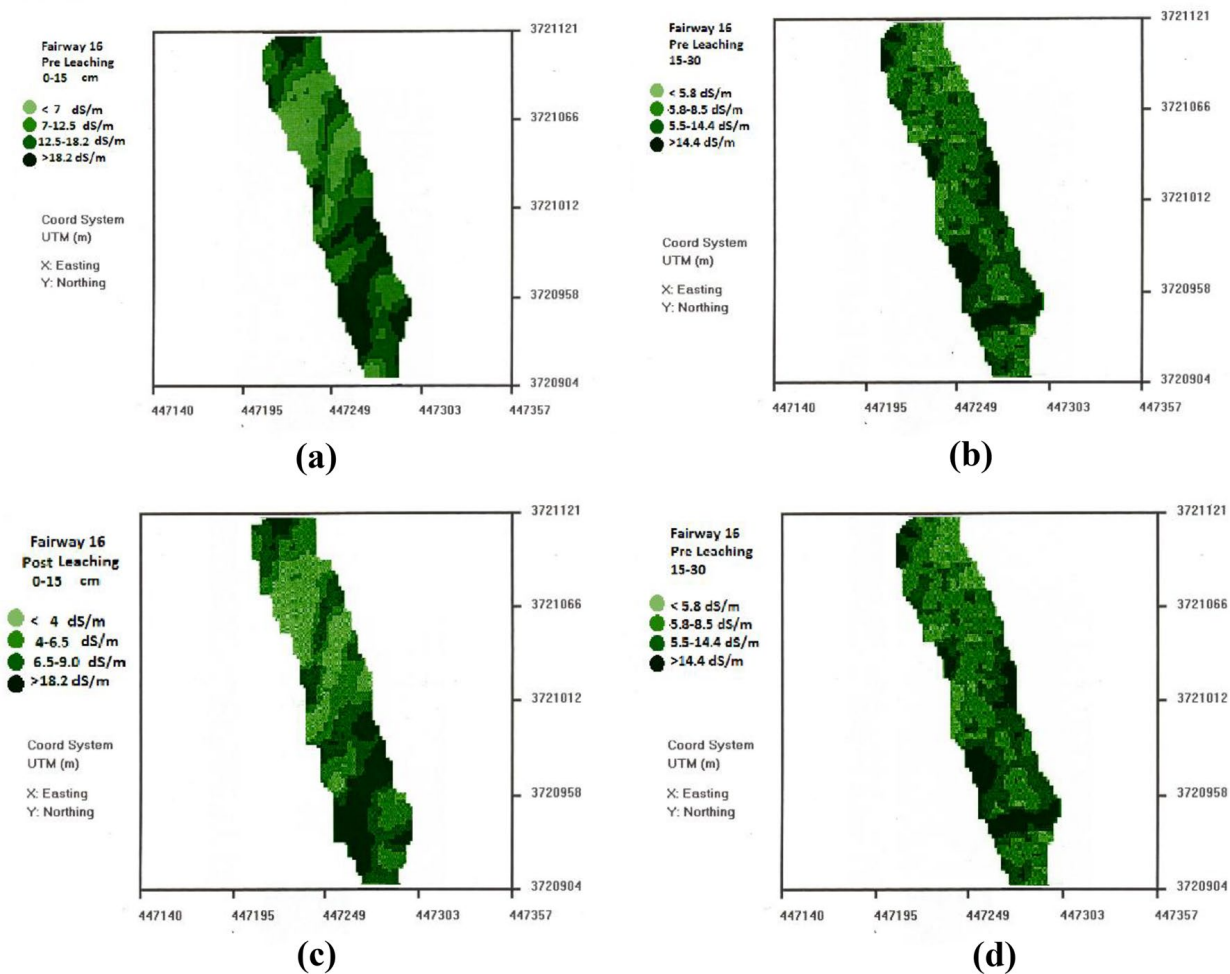
Pre-leaching (**a**) 0–15 cm, (**b**) 15–30 cm and post leaching (**c**) 0–15 cm, (**d**) 15–30 cm salinity maps (ECe) for fairway 16 created using ESAP.

As shown in Fig. 1a in the salinity map for fairway 16, there was considerable variation in salinity in the 0–15 cm depth with about 50% of the fairway above EC_e_ = 5 dS m^−1^ and an extensive area above EC_e_ = 18 dS m^−1^ a value where the turf would be severely impacted. The salinity map for 15–30 cm depth (Fig. 1b) shows reduced salinity relative to the soil surface, suggesting a lack of sufficient leaching with current irrigation regime, consistent with the measured water applications being below ET. At both depths the salinity is greater in the lower (southern) portion of the fairway suggesting the potential for differential leaching in these two regions.

**Figure 2:**
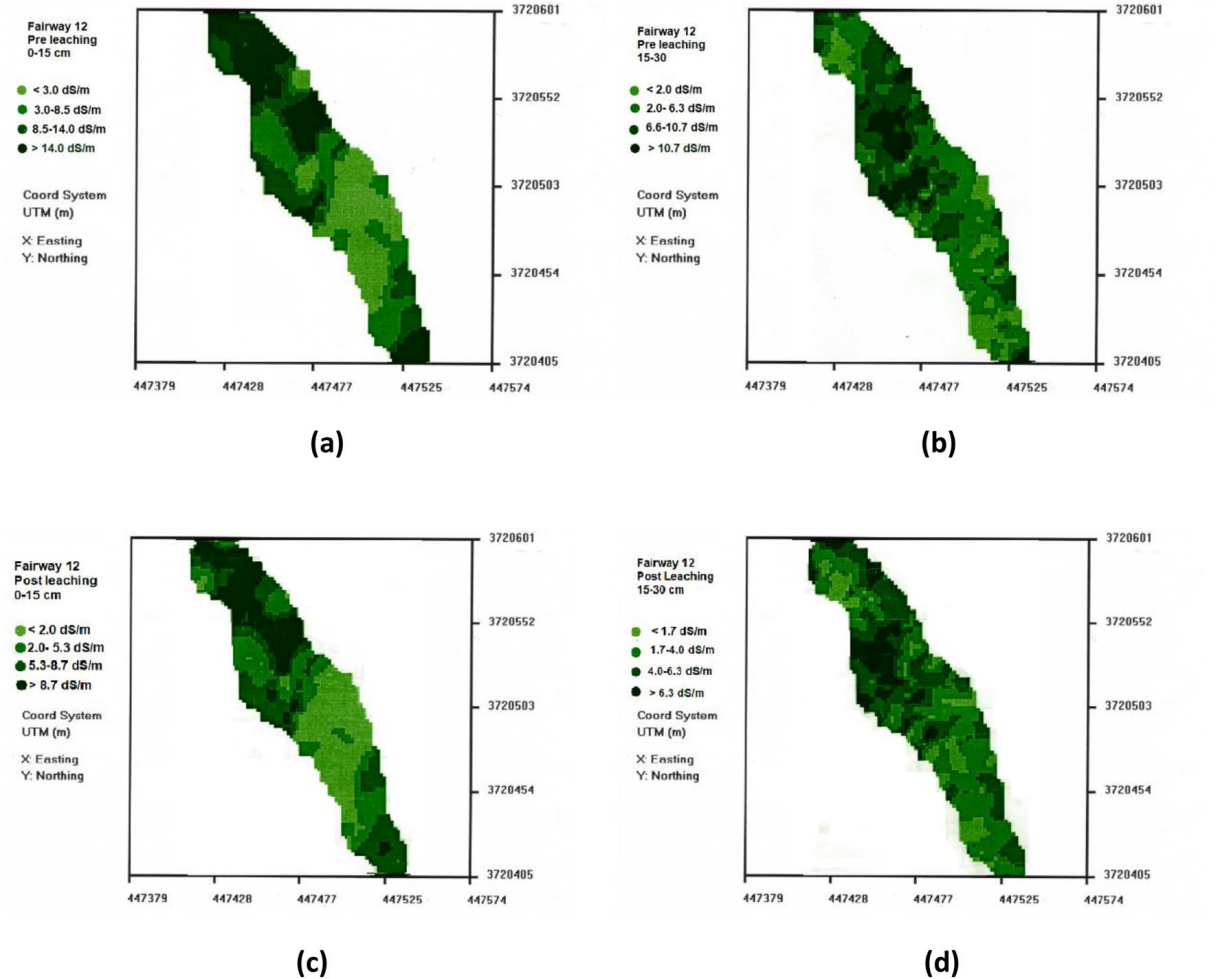
Pre-leaching (**a**) 0–15 cm, (**b**) 15–30 cm and post leaching (**c**) 0–15 cm, (**d**) 15–30 cm salinity maps (ECe) for fairway 12 created using ESAP.

Based on the EC_e_ map generated for fairway 12, we calculate that 10% of the fairway had an EC_e_ greater than 6.9 dS m^−1^ at 15–30 cm depth thus less than 10% had salinity levels that would cause either significant reduction in growth or unacceptable turf appearance. In this fairway most of the poor turf condition is attributed to shallow soil with insufficient water holding capacity, resulting in likely water stress between irrigations. The detailed EC_e_ map based on the EM survey is shown in Fig. 2a,b for 0–15 and 15–30 cm depth, respectively, providing information on the spatial distribution of the salinity.

### Post leaching salinity

The post leaching maps of fairway 16 are shown in Fig. 1c,d for 0–15 and 15–30 cm depths, respectively. As with the pre-leaching maps there was high field variability regions of high salinity. Over 50% of the fairway still had EC_e_ values above 6.5 dS m^−1^ at 0–15 cm depth.

**Figure 3:**
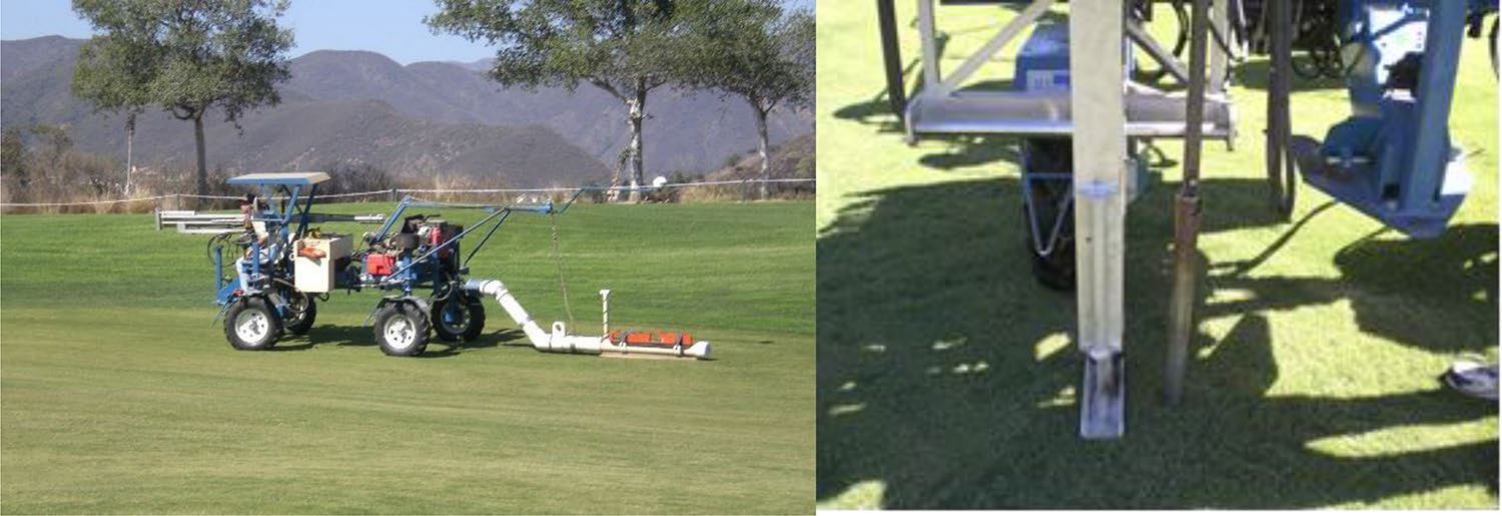
(**a**) EM 38 remote sensing field vehicle with EM38 unit positioned for EC measurements at the site. (**b**) Close up of soil coring instrument mounted to field vehicle.

The post leaching map of fairway 12 is given in Fig. 2c,d, for 0–15 and 15–30 cm depth respectively. Only small portions of fairway 12 (upper portions of Fig. 3c) were still adversely affected by salinity exceeding EC_e_ > 8.7 dS m^−1^.

### Infiltration rates in fairway 12

The measured infiltration rate based on the Guelph permeameters varied between 3.5 to 264 mm h^−1^ with a mean value of 76 mm h^−1^ at the soil surface and between 1.5 and 0.3 with a mean value of 1.11 mm h^−1^. Most of the field is estimated to have an infiltration rate lower than the sprinkler application rate 17 mm h^−1^. There was no evidence of surface runoff during irrigation.

## Discussion

The 15–30 cm salinity on Fairway 16 before reclamation was lower than the 0–15 cm salinity levels based on both the salinity survey and the soil cores. This suggests either preferential flow/by pass in the near surface environment and or under irrigation. The pre-leaching salinity levels in fairway 16 are high enough to cause a decrease in visual quality of Bermuda grass hence the decision to attempt reclamation by leaching the soil. Bermuda grass has a salinity threshold level of EC_e_ 6.9 dS m^−1^ above which there is loss of vegetative growth^21^ but not until EC_e_ 15 dS m^–1^ does the grass drop below a rating of 6 for visual quality^22^ and growth is reduced by 50%^23^. Xiang et al.^24^, documented a drop in live green cover for several Bermuda grasses from greater than 80% cover at EC below 15 dS m^−1^ to less than 50% cover at EC above 15 dS m^−1^^25^.

The soil salinity in fairway 16 after reclamation leaching with 153 mm of water decreased by 43% for the 0–15 cm depth and by 58% for the 15–30 cm depth after application. This suggests inefficient leaching because the expected decrease in salinity would be predicted to be much greater than observed, especially for the 0–15 cm depth. We used the relations given by Keren and Miyamoto^26^ for intermittent ponding but added correction for the EC of the irrigation water The calculated depth of leaching water divided by soil depth, 2.28 dS m^−1^ for 0–15 cm and 2.88 for 15–30 cm depth, after correction for EC of irrigation water, should have resulted in salinity level decreases of 81% and 76% respectively or mean EC_e_ values of 2.28 and 2.88 dS m^−1^, respectively.

The limited effect of water application on soil salinity is attributed to water bypass. Macropore flows of leaching water result in water and solute bypass in the saline soil matrix, resulting in reduced leaching efficiency^27, 28^. This hypothesis is reinforced by the relatively poor leaching in the 0–15 cm depth as compared to leaching in the 15–30 cm depth, as well as the use of mechanical aeration paradoxically utilized to improve water penetration. Macropores lead to increase preferential flow, infiltration rates and to decrease surface runoff^29^. Mechanical aeration creates large macropores that restrict surface leaching as a result of lower contact time in the root zone.

Post leaching, the mean salinity level of the fairway was well below the threshold level at which Bermuda grass would be affected^25^. However, the limitation of using mean values is indicated by the EC_e_ maps for fairway 16 (Fig. 1c,d), as many regions of the fairway were still adversely affected by salinity at both 0–15 and 15–30 cm depth. The salinity maps were generated by dividing the calculated EC_e_ values into quartile salinity distributions. Comparing Fig. 1a, (pre-leaching) and Fig. 1c (post leaching) for the 0–15 cm depths, we note the striking similarity of the spatial distribution or salinity patterns. The salinity levels were reduced in all areas, but the higher salinity areas remained higher after leaching. In this instance we see the potential benefit of site-specific management (leaching) but unfortunately the current irrigation system could not be easily modified to use the leaching water more effectively.

Fairway 12 demonstrated a similar response to leaching as fairway 16 did to leaching. The 37.5% and 36.9% reduction in average salinity for the 0–15 cm and 15–30 cm soil depths, respectively were again much less than expected (82% and 73%) based on applied water^26^, suggesting as was the case in fairway 16, that water bypass limited salinity leaching^27, 28^, especially in the 0–15 cm depth. Fairway 12 received more leaching water than fairway 16 and thus should have been more extensively leached. The mean salinity levels are below the threshold level at which Bermuda grass would be affected by salinity. There was no evidence of surface runoff during irrigation. It seems reasonable that some water redistribution might occur during irrigation, but this is not sufficient to explain the lack of complete leaching that would be expected for the 0–15 cm depth after 264 mm of leaching water according to relations given by Keren and Miyamoto^26^, corrected for irrigation water salinity. The expected salinity of EC_e_ = 1.51 dS m^−1^, would be in equilibrium with the irrigation water salinity. We did not observe surface runoff despite the water application rate exceeding the permeameter measured infiltration rates. Also, there was no evidence of surface erosion despite slopes exceeding 5% in some areas. All permeameter values were below the irrigation rate at the 15–30 cm depth. As with fairway 16, we can only consider that concurrent aeration practices resulted in large macropore flow. The poor turf quality in this fairway is likely the result of water bypass at the surface resulting in intermittent water stress between irrigation events rather than elevated salinity.

## Conclusion

Soil salinity is temporally variable and exhibits a complex pattern in the field that cannot be easily captured by conventional soil sampling. Traditional assessment of soil salinity in the field scale is very time consuming and labor dependent on both laboratory and field studies. The sensing equipment is demonstrated to provide detailed and relatively rapid mapping, but accurate calibration is not always simple. Accurate mapping requires site specific calibration and may still fail to provide a good calibration between soil samples and sensor readings. In this study, we briefly describe the current best approaches for calibration and evaluate the methodology for a field study with EM survey and data actuation for grass landscapes. In regions with water shortages, such as Southern California, USA, the spatial distribution of salinity is extremely important for reducing the amount of leaching water where reclamation is needed. In these areas water is scarce and leaching water expensive. Site-specific leaching on landscapes can reduce irrigation and leaching costs but is limited as most current irrigation systems do not have the flexibility to preferentially leach portions of a field or golf fairway. This study focused on creating and interpreting salinity maps before and after leaching in a landscape vegetation (golf course) that was considered to have salinity problems with different water applications on two different fairways. Efficacy of leaching was evaluated and likely factors impeding salinity reduction identified, specifically aeration. Aeration improved water penetration but ironically impeded salt leaching as it caused water to bypass the shallow soil depths. The analysis enables management changes to improve turfgrass quality. In one instance the likely cause of poor turf quality is attributed to low water holding capacity of a shallow soil and insufficient irrigation frequency. In future reclamation studies, the impact of soil disturbance (tillage and aeration) on salinity leaching should be further examined especially in light of the need to limit quantities of water used for leaching. Increasing water infiltration may not efficiently reduce soil salinity.

## Materials and methods

### Site description and irrigation water quality

This study was carried out in Dove Canyon Golf Course in Trabuco Canyon, California, US.

The main grass species in the fairways at the golf course is bermuda grass (*Cynodon dactylon* L.). Treated municipal wastewater has been used as irrigation water source. As analyzed, the water has an EC_w_ of 1.32 dS m^−1^ and a SAR (sodium adsorption ratio defined as Na^+^[(Ca^2+^ + Mg^2+^)/2] where units are mmol_c_ L^−1^) of 4.2 with cation composition of Na^+^  = 7.65, Ca^2+^  = 3.79, and Mg^2+^  = 4.51, where units are mmol_c_ L^−1^. This salinity level is acceptable for bermuda grass irrigation under good salinity management practices. Salinity tolerance of bermuda grass (*Cynodon* spp.) can vary greatly among different bermudagrass cultivars or phenotypes^27^. Bermuda grass is reported to be moderately salt sensitive (EC_e_ 3–6 dS m^−1^) or moderately tolerant (6–10 dS m^−1^) depending on the cultivar examined^28^. Grieve et al.^21^, list the threshold EC at which growth decreases as 6.9 dS m^−1^.

Fairways 12 and 16 were evaluated for salinity effects as both had visible signs of stress and reduced vegetative cover. Sprinklers used for irrigation at the golf course had a theoretical application rate of 19.1 mm h^−1^. Based on catch-can tests, the measured application rate was 17.2 mm h^−1^, hence all water applications based on irrigation time were corrected by a factor of 0.90.

### Salinity survey and sampling design

Salinity assessment was carried out by means of a mobile unit (Fig. 3a) with an EM38 instrument positioned vertically and horizontally in order to read both conductivity data at the same time (Fig. 3b). The EM_v_ (vertical) reading penetrates to a depth of approximately 1.2–1.5 m, while the EM_h_ (horizontal) reading penetrates to approximately 0.60–0.75 m^28^. The ESAP-95 program^18–20^ was used to process the EM38 survey data and generate sampling plans for calibration using saturation extracts from soil cores. The algorithm in this program selects a limited set of calibration sites with desirable spatial and statistical characteristics based on analysis of EC_a_ values and survey site location information using response surface design techniques^29^. Critical evaluation of response surface design and a unified sampling and modeling strategy for predicting soil property information from spatially referenced sensor data has been presented in detail by Lesch^19^. Thus, after collection and preliminary analysis of the electromagnetic induction signal data we collected sample soil cores from various locations within the field as indicated by the ESAP software. The accuracy of the salinity survey thus depends on the accuracy and precision used in both the survey and profile data acquisition processes, in addition to the correlation between these two data sets.

### Soil sampling and mapping

**Figure 4:**
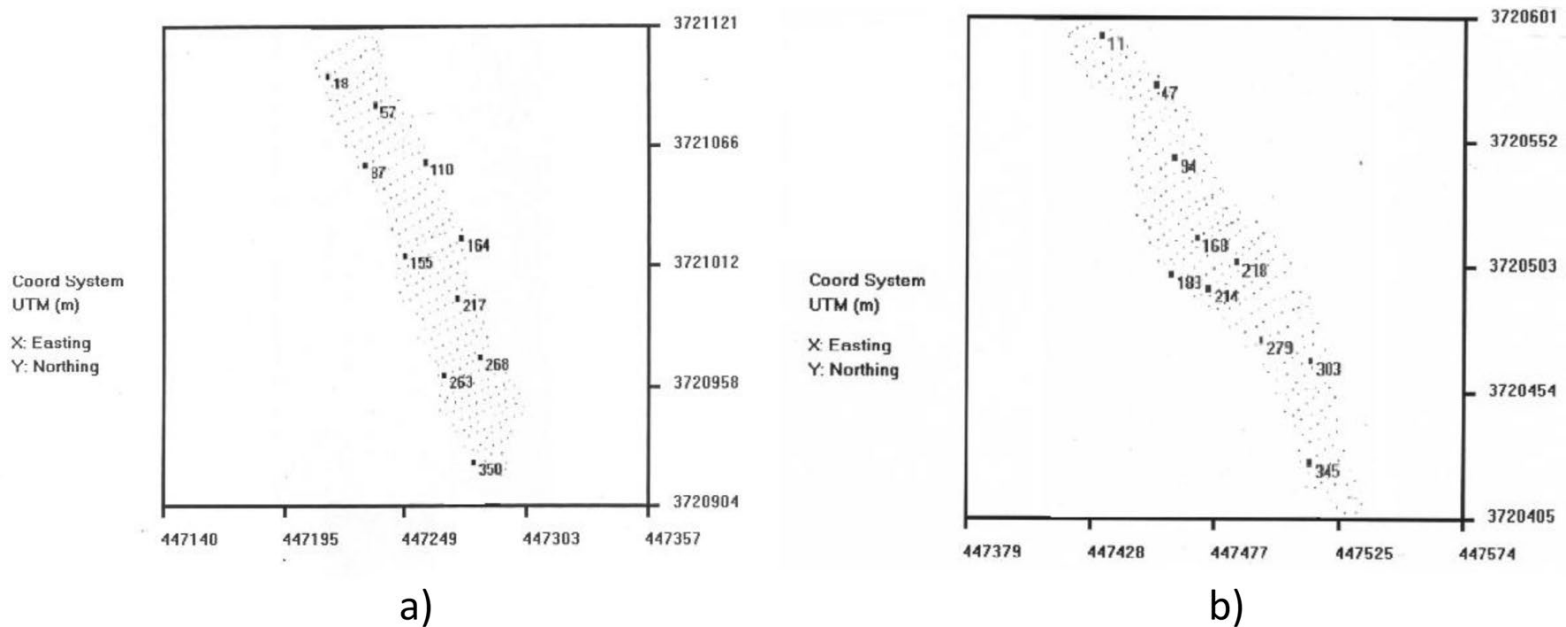
Soil Sampling design for (**a**) fairway 16 and (**b**) fairway 12 created via ESAP software.

The *ESAP* software package identifies the optimal locations for soil sample sites from the EC_a_ survey data. These sites are selected based on spatial statistics to reflect the observed spatial variability in EC_a_ survey measurements. Generally, 6 to 20 sites are selected depending on the level of variability of the EC_a_ measurements for a site. The optimal locations of a minimal subset of EC_a_ survey sites are identified to obtain soil samples^15^. According to the EM38 readings and ESAP program outputs, 60 cm deep column samples, or until bedrock was reached) were taken from the 10 calibration points per fairway (Fig. 4). The columns were divided into 0–15, 15–30, 30–45 and 45–60 cm depths and then analyzed for water content, saturation percentage, EC_e_ and pH_e_ and major cation composition according to standard methods^31^ and cations by PerkinElmer Optima 3300DV.

ICP OES (inductively coupled plasma optical emission spectroscopy-PerkinElmer Corp, Waltham, MA, USA). Salinity maps were created with the ESAP-SaltMapper program using output prediction data files created via the ESAP-Calibrate module. Utilizing ESAP-SaltMapper Raster Image Map main menu, data interpolation was performed using on-screen interpolation controls. This step includes specifying map variable (color), kernel size, cut-off levels, and scale factor. Inverse distance weighting (IDW) was used as interpolation method. This procedure was repeated at the same sites for post leaching salinity analysis. A paired t-test was applied to statistically assess the changes between the pre-versus post-leaching salinity levels. At selected sites in each fairway, we utilized Guelph permeameters to measure hydraulic conductivity using the site irrigation water.

The soil sampling design shown in Fig. 4a,b for fairway 16 and 12 respectively were generated by ESAP based on the initial EM-38 survey.

### Leaching

The initial extra water event for soil reclamation was initiated on 7/16–7/19 (4 nights and 3 days). Sprinklers were run on 20 min cycles, with at least a 20-min period in between each cycle. The calculated applied water for the 7/16–7/19 leaching event was 152.8 mm. In addition, 11.7 mm of water were applied during this period to compensate for ET. Leaching water was the same source as irrigation water; municipal treated wastewater. In addition to these EM 38 measurements, we also collected soil samples and infiltration data for fairway 12 after leaching with an additional 101 mm of water.

### Field survey and salinity mapping

The salinity survey was divided into two parts, pre-leaching, and post-leaching surveys. In the pre-leaching survey, EM38 readings (both EM_v_ and EM_h_ data) were saved simultaneously with GPS data on a data logger mounted on the vehicle (Fig. 3a), thus each reading was associated with spatial data. After the transect readings and GPS data were collected, The ESAP RSSD module was run to develop the sampling design. The program determined 10 sampling points as shown in Fig. 4 for fairway 16 with GPS coordinates. We navigated to each sampling coordinates and collected soil cores by means of a hydraulic soil corer attached to the vehicle (Fig. 3b). The system had a metallic column to break down the soil’s mechanic resistance and a plastic column sleeve inside to take the soil samples. Totally, 10 plastic columns with a height of up to 60 cm were taken to the lab from each fairway.

The results of the soil analyses and the EM data were run on ESAP module CALIBRATE and then the program generated outputs via ESAP SALTMAPPER module as EC_e_ salinity maps and ASCII data. We utilized only the 0–15 and 15–30 cm soil data for analysis and interpretation. At many locations soil depth was less than 45 cm thus comprehensive data on the spatial distribution of salinity at 30–45 and 45–60 cm depth could not be obtained. Restriction of the analysis to the 0–30 cm depth is not a problem because turfgrass roots are concentrated in the 0–30 cm depth^30, 31^.These same processes including EM38 transect readings and use of ESAP- SALTMAPPER were carried out after leaching (post-leaching).
